# Co-Crystals of Resveratrol and Polydatin with L-Proline: Crystal Structures, Dissolution Properties, and In Vitro Cytotoxicities

**DOI:** 10.3390/molecules26185722

**Published:** 2021-09-21

**Authors:** Yijie Lou, Kaxi Yu, Xiajun Wu, Zhaojun Wang, Yusheng Cui, Hanxiao Bao, Jianwei Wang, Xiurong Hu, Yunxi Ji, Guping Tang

**Affiliations:** 1 First Clinical Medical College, Zhejiang Chinese Medical University, Hangzhou 310053, China; leochwe@126.com (Y.L.); 18167156759@163.com (X.W.); 15027059040@163.com (Z.W.); a741542704@163.com (Y.C.); 2 Department of Chemistry, Zhejiang University, Hangzhou 310028, China; yukaxi@zju.edu.cn (K.Y.); 3170103016@zju.edu.cn (H.B.); wjw126hz@zju.edu.cn (J.W.); huxiurong@zju.edu.cn (X.H.)

**Keywords:** resveratrol, polydatin, L-proline, pharmaceutical co-crystal, crystal structure, solubility and dissolution rate

## Abstract

Resveratrol (RSV) and polydatin (PD) have been widely used to treat several chronic diseases, such as atherosclerosis, pulmonary fibrosis, and diabetes, among several others. However, their low solubility hinders their further applications. In this work, we show that the solubility of PD can be boosted via its co-crystallization with L-proline (L-Pro). Two different phases of co-crystals, namely the RSV-L-Pro (RSV:L-Pro = 1:2) and PD-L-Pro (PD:L-Pro = 1: 3), have been prepared and characterized. As compared to the pristine RSV and PD, the solubility and dissolution rates of PD-L-Pro in water (pH 7.0) exhibited a 15.8% increase, whereas those of RSV-L-Pro exhibited a 13.8% decrease. A 3-(4,5-dimethylthiazol-2-yl)-2,5-diphenyltetrazolium bromide (MTT) assay of pristine RSV, PD, RSV-L-Pro, and PD-L-Pro against lung cancer cell line A549 and human embryonic kidney cell line HEK-293 indicated that both compounds showed obvious cytotoxicity against A549, but significantly reduced cytotoxicity against HEK-293, with PD/PD-L-Pro further exhibiting better biological safety than that of RSV/RSV-L-Pro. This work demonstrated that the readily available and biocompatible L-Pro can be a promising adjuvant to optimize the physical and chemical properties of RSV and PD to improve their pharmacokinetics.

## 1. Introduction

**Figure 1:**
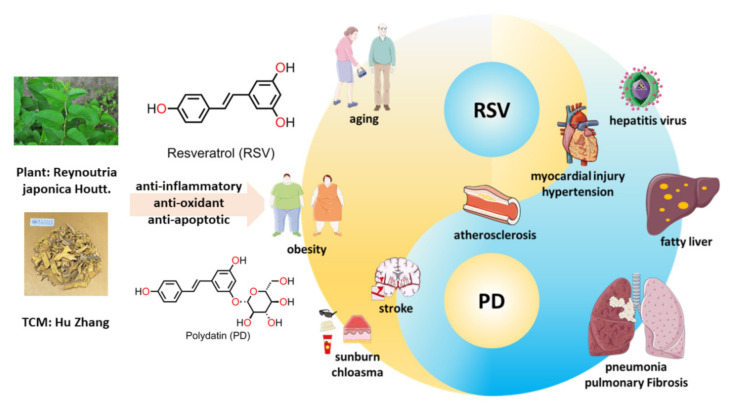
The structures of RSV and PD and their shared and separate clinical roles.

Polydatin (PD) is a natural precursor and the glucose derivative of trans-3,5,4’-trihydroxystilbene (resveratrol; denoted as RSV) [1]. RSV and PD are extractives of radix or rhizoma of Polygonum cuspidatum. By sharing the RSV moiety, RSV and PD have many similar pharmacological effects. As the main active ingredients of traditional Chinese medicine (TCM), they show good therapeutic properties, such as anti-inflammatory, anti-oxidant, and anti-apoptotic effects [1]. RSV exhibits the advantages of anti-aging, weight loss, and skin protection, while PD performs better in anti-virus and anti-fibrosis activities, and in the promotion of lipid metabolism [2,3,4,5]. Therefore, both RSV and PD are used as anti-oxidants, and complement each other in clinical treatment. Various studies have confirmed that RSV and PD are widely used to treat several chronic diseases, such as atherosclerosis, pulmonary fibrosis, diabetes, cerebral ischemia, steatohepatitis, and various carcinomas (Figure 1) [3,5,6,7,8,9,10,11].

According to the biopharmaceutics classification system (BCS), RSV and PD are classified as category II due to their low solubility and high intestinal permeability [11,12,13]. As both are orally administered, their bioavailability is often hindered by their poor water solubility and stability [13,14,15,16,17]. Since there is no issue with the permeability of RSV and PD, increasing their water solubility and/or dissolution rate should be a promising method to obtain satisfactory bioavailability. Several relevant methods have been developed to improve the solubility, oral bioavailability, and circulation time of RSV and PD, such as the formation of dispersed solids with Mg(OH)_2_, encapsulation by liposomal nanoparticles, and pH dual-sensitive block polymer PEG-P(PBEM-co-DPA) [13,18,19,20,21,22,23].

In the last decade, co-crystallization has emerged as a convenient entry to increase the solubility of the otherwise less-insoluble drugs [24]. Co-crystallization alters the mechanical stability, hygroscopicity, and ultimately the bioavailability by switching the molecular interaction between components to generate a unique structure composition [25,26]. However, there are few studies on improving the solubility and bioavailability of RSV and PD through co-crystallization, though RSV polymorphs and its 4-aminobenzamide, isoniazid, and L-proline (L-Pro) co-crystals have been previously revealed [27,28].

It is well-known that due to the presence of hydrogen-bonding donor and acceptor sites in the molecule, L-Pro has a good potential to form co-crystals, and has negligible biological toxicity [29,30,31,32,33]. In this work, we report L-Pro co-crystals of RSV and PD, namely RSV-L-Pro and PD-L-Pro, prepared under different synthetic conditions. RSV-L-Pro and PD-L-Pro were characterized to exhibit stoichiometric ratios of 1:2 and 1:3 between the drug and L-Pro. We found that upon co-crystallization with L-Pro, the formed RSV-L-Pro exhibited a reduced solubility and dissolution rate as compared to pristine RSV. By contrast, those of PD-L-Pro were marginally enhanced relative to the pristine PD. A 3-(4,5-dimethylthiazol-2-yl)-2,5-diphenyltetrazolium bromide (MTT) assay for RSV, PD, RSV-L-Pro, and PD-L-Pro against lung cancer cell line A549 and human embryonic kidney cell line HEK-293 showed that all these compounds had no obvious cytotoxicity to HEK-293, but were toxic to A549 after culturing the cells for an extended period of 24 h. Meanwhile, PD-L-Pro had a better biosafety performance than RSV-L-Pro.

## 2. Results and Discussion

### 2.1. Synthesis and Characterizations

**Figure 2:**
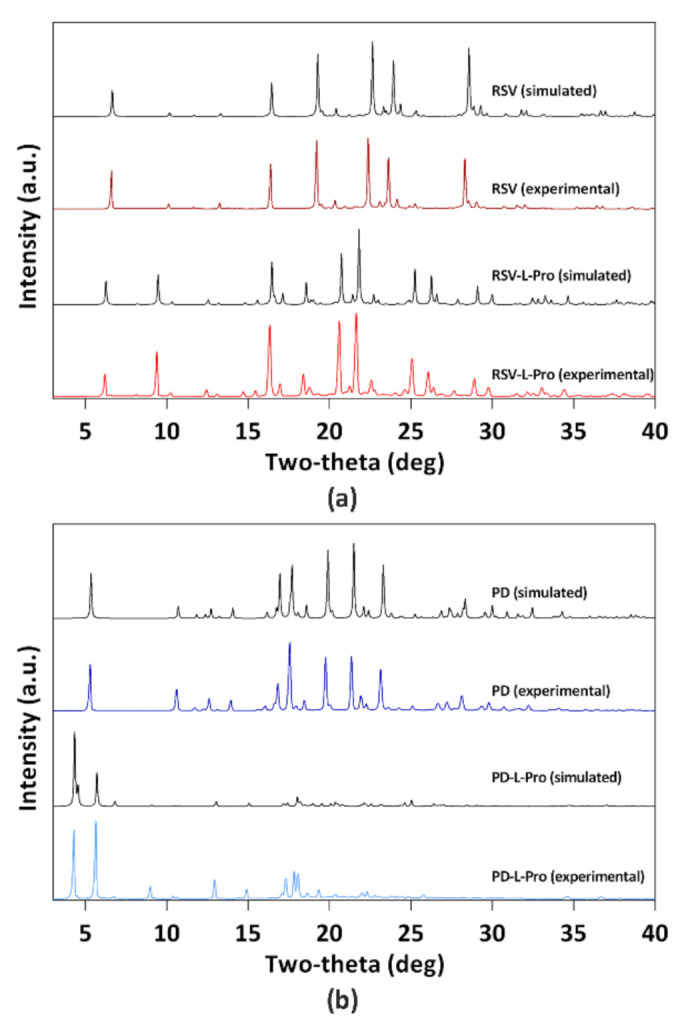
PXRD patterns of RSV and RSV-L-Pro (**a**) and PD and PD-L-Pro (**b**) showing a good agreement between the experimental results and those simulated from single-crystal diffraction data, indicating high phase purity of all the four complexes, and thus the successful formation of new phases of RSV-L-Pro and PD-L-Pro.

The RSV-L-Pro and PD-L-Pro both were prepared as single crystals from a molar equivalent of RSV and PD with L-Pro in EtOH at 60 °C. We used powder X-ray diffraction (PXRD) to perform solid-state characterization of RSV, PD, RSV-L-Pro, and PD-L-Pro (Figure 2 and Table S1). As depicted in Figure 2, the experimental PXRD of all four complexes were in decent agreement with those simulated from single-crystal X-ray diffraction data (as will be discussed later), indicating their high phase purities. The PXRD pattern of RSV-L-Pro/PD-L-Pro also was drastically different from that of RSV/PD, an indication of new phase formation.

**Figure 3:**
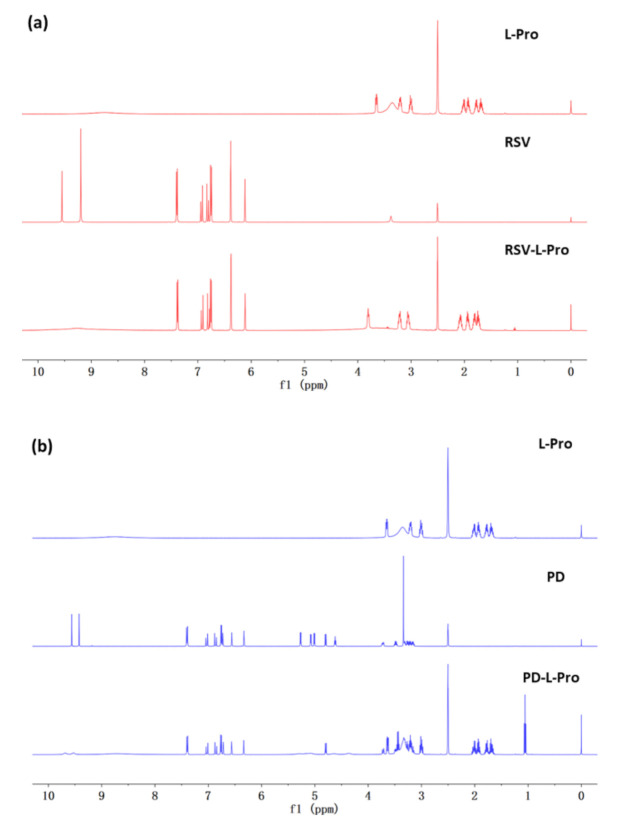
The ^1^H NMR spectra of L-Pro, RSV, and PD, as well as for their respective co-crystals RSV-L-Pro (**a**) and PD-L-Pro (**b**).

The ^1^H nuclear magnetic resonance (NMR) spectra recorded for the crystalline samples of L-Pro, RSV-L-Pro and PD-L-Pro dissolved in DMSO-*d*_6_ (Figure 3 and Figures S1–S3) confirmed their identity, purity, and the stoichiometric ratios between the drug and L-Pro (1:2 for RSV-L-Pro and 1:3 for PD-L-Pro).

**Figure 4:**
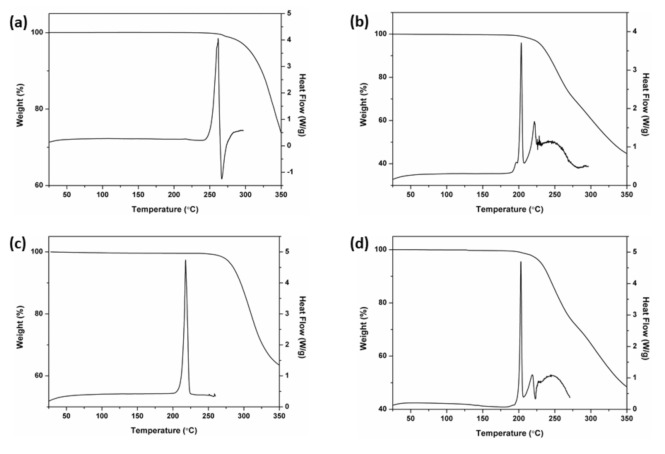
TGA-DSC for the L-Pro (Figure S4), RSV (**a**), RSV-L-Pro (**b**), PD (**c**), and PD-L-Pro (**d**).

Thermogravimetric analysis (TGA) coupled with differential scanning calorimetry (DSC) were used to characterize RSV-L-Pro and PD-L-Pro. As shown in Figure 4b, there was a sharp and strong endothermic peak at 227.6 °C for RSV-L-Pro. Combined with the subsequent chaotic DSC endothermic curve, this indicated that the sample gradually decomposed after melting. This was in contrast to what was observed for the pristine RSV, which was stable up to 252.8 °C (Figure 4a). Similar conclusions were also drawn after comparing PD-L-Pro (Figure 4d) and PD (Figure 4c). The EtOH-free powder of PD-L-Pro exhibited a strong endothermic peak at 225.7 °C, and the sample also gradually decomposed after melting.

### 2.2. Crystal Structure Analysis

**Figure 5:**
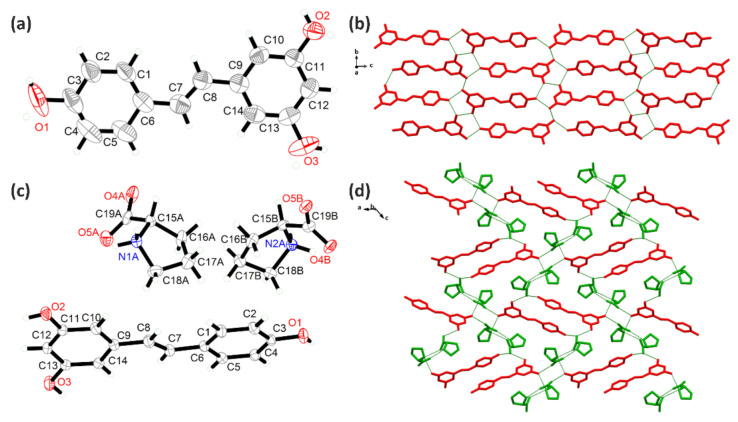
The asymmetric unit of RSV (**a**) and RSV-L-Pro (**c**), as well as their hydrogen-bonded network (**b**,**d**).

The RSV and RSV-L-Pro crystallized in the monoclinic *P*2_1_/*c* and orthorhombic *P*2_1_2_1_2_1_ space groups (Table 1). The RSV (Figure 5a,b) molecules shaped an explicit 2D network within the *bc* plane due to the hydrogen bonds. Meanwhile, the asymmetric unit of RSV-L-Pro contained one RSV and two PRO molecules (Figure 5c). In RSV-L-Pro, the L-Pro moiety also was in the zwitterionic form, wherein the H atoms from the –COOH group migrated to the –NH– to yield the –[COO]^−^ and –[NH_2_]^+^– pair [34]. The –[COO]^–^ also was in a conjugated form, serving as the H-bonding acceptors. As a result, due to the sufficient H donors and acceptors from three –OH groups of the RSV, two pairs of –[COO]^−^ and –[NH_2_]^+^– in L-Pro, extensive H-bonding interactions (Table S2) were available. The connections between RSV and PRO shaped adjacent chains along the *a* axis, while the N–H∙∙∙O hydrogen bonds between PRO molecules along the *b* axis formed a three-dimensional (3D) network (Figure 5d).

**Figure 6:**
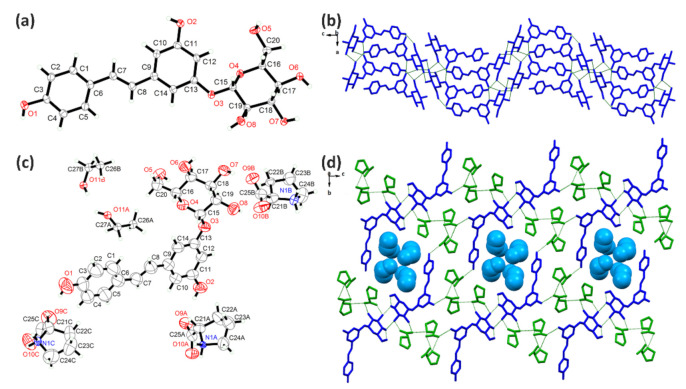
The asymmetric unit of PD (**a**) and PD-L-Pro (**c**), as well as their hydrogen-bonded network (**b**,**d**).

The PD and PD-L-Pro compounds crystallized in the orthorhombic *P*2_1_2_1_2_1_ and monoclinic *C_2_* space groups (Table 1). The molecule of PD had an additional glycosidic group compared to RSV, which gave it a bulkier size with more –OH groups (Figure 6c). Therefore, there were more hydrogen bonds on the crystal structure of PD (Figure 6a,b), which shaped a 2D network within the *ac* plane. Upon crystallization with L-Pro, the PD-L-Pro had one PD molecule and three PRO molecules (also in zwitterionic form), as well as two crystalline EtOH solvates in the asymmetric unit. Interestingly, in PD-L-Pro, two PD molecules formed a dimer, and three PRO molecules shaped a trimer due to the presence of rich H-bonding interactions. Furthermore, one PD dimer and two PRO trimmer shaped large rings, and with the associations among PD and PRO molecules, a 3D H-bonded network was formed that featured 1D channels along the *a* axis to host the crystalline EtOH solvates (Figure 6d).

### 2.3. Hirshfeld Surface Analysis of RSV, PD, and Their Co-Crystals with L-Pro

**Figure 7:**
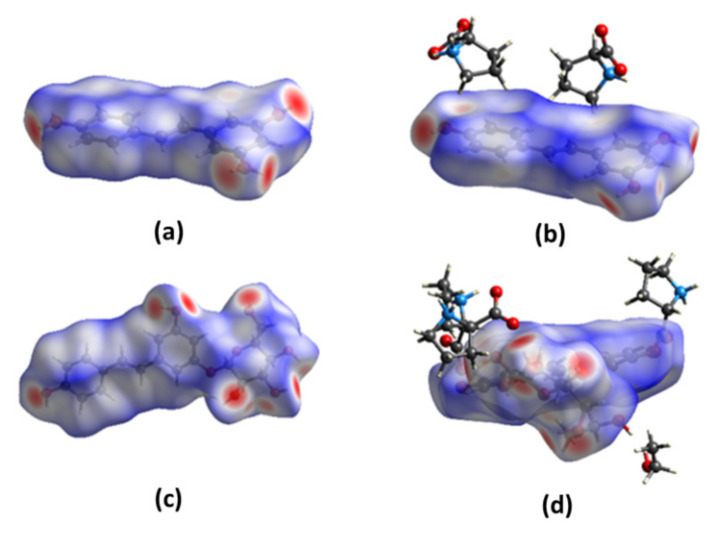
Hirshfeld surfaces for the RSV (**a**), RSV-L-Pro (**b**), PD (**c**), and PD-L-Pro (**d**).

To assess and compare the intermolecular interactions in the four compounds, Hirshfeld surface (HS) and two-dimensional (2D) fingerprint maps were generated using the program Crystal Explorer [35]. We found detailed atom-pair close contacts of major molecules, including O···H/H···O, C···H/H···C, and H···H contacts. The deep-red spots on the Hirshfeld surface reveal the shortest O···H/H···O interactions (Figure 7). Overall, the HS of RSV-L-Pro and PD-L-Pro differed from each other in shape, reflecting the different weights of intermolecular contacts.

**Figure 8:**
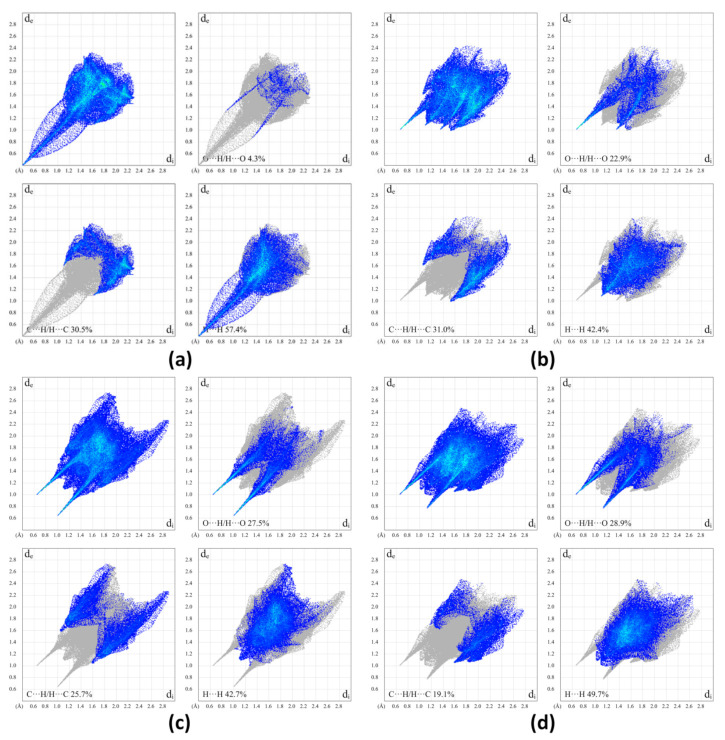
Two-dimensional fingerprint plots of RSV (**a**), RSV-L-Pro (**b**), PD (**c**), and PD-L-Pro (**d**).

Intermolecular interactions appeared as discrete spikes for PD and PD-L-Pro, while shown as a single spike for RSV and RSV-L-Pro in the 2D fingerprint plots. The quantitative analysis (Figure 8) showed that the H···H and O···H/H···O interactions were almost equal contributors to the total HS for four structures as compared to C···H/H···C and other contacts, represented by the large surfaces (blue) in the fingerprint plots. It is worth noting that the connections between RSV and L-Pro in RSV-L-Pro had a higher contribution of O···H/H···O interactions (22.9%) than RSV (4.3%), while the L-Pro molecules in PD-L-Pro had a small increased contribution of O···H/H···O (27.5%) to PD (28.9%).

### 2.4. Solubility Analyses of RSV, RSV-L-Pro, PD, and PD-L-Pro

**Figure 9:**
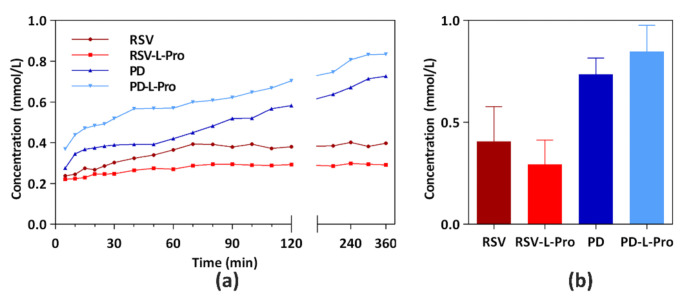
(**a**) A summary of the solubility profile and (**b**) solubility data upon equilibrium for 24 h of RSV, PD, and the crystals of RSV-L-Pro and PD-L-Pro in H_2_O at pH 7.0. Data are expressed as average values from three replicates.

As shown in Figure 9, PD-L-Pro reached dissolution equilibrium in 4 h, with a 15.8% solubility increase as compared to PD. By contrast, RSV-L-Pro exhibited a dissolution equilibrium time of 1.5 h with a 13.8% solubility decrease. It can be seen that L-Pro played a positive role in improving the solubility of PD. In contrast, L-Pro contributed negatively to the solubility of RSV, and similar observations were also made using nicotinamide (NA) and isonicotinamide (INA) as the adjuvant [27]. Since PD carries one additional glucoside than RSV, we believe that it contributed to the enhanced solubility as compared to RSV by forming abundant hydrogen bonds in the aqueous phase. In addition, the formation of channel-like structures in PD-L-Pro may have facilitated the quick diffusion of water molecules into the crystal lattice to accelerate the dissolution of the co-crystal.

### 2.5. MTT Assays

**Figure 10:**
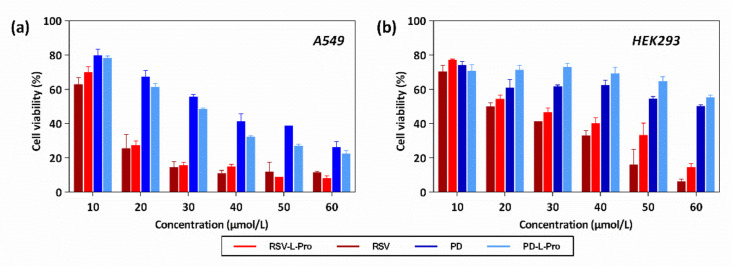
The cell viability data of RSV, PD, and the crystals of RSV-L-Pro and PD-L-Pro against human lung cancer cell line A549 (**a**) and human kidney cell line HEK-293 (**b**) upon culturing for 24 h.

RSV and PD have a wide range of pharmacological effects and are used clinically as complementary therapies [3,5,6,7,8,9,10,11]. The main method of use is oral administration. This creates higher demands for the safety of RSV and PD co-crystals. At the same time, it has been reported that both RSV and PD exhibited certain anti-tumor activities, such as for lung cancer [36,37,38]. RSV and PD are capable of inducing the apoptosis of lung cancer A549 cells. To further evaluate the biosafety of RSV, PD, RSV-L-Pro, and PD-L-Pro, as well as their potential as anti-cancer agents, we evaluated the biological activities of the pristine RSV, PD, RSV-L-Pro, and PD-L-Pro against the proliferation of the A549 cancer cell line (murine origin) and HEK-293 (human origin) cell line using the 3-(4,5-dimethylthiazol-2-yl)-2,5-diphenyltetrazolium bromide (MTT) assay. In the comparison of RSV and PD, the inhibitory effect of RSV against A549 was more obvious than that of PD (Figure 10a). However, RSV showed greater biological toxicity to HEK-293. The cytotoxicity of PD against HEK-293 was nearly negligible (Figure 10b). Therefore, from the perspective of biological safety, PD is safer than RSV. Although it has certain anti-cancer activity, it cannot compete with the first-line anti-cancer drugs, but can be used as an adjuvant drug instead. The anti-cancer activity and cell safety of PD-L-Pro had a certain increase compared with PD. Compared with RSV, RSV-L-Pro had a nearly identical cytotoxicity against A549, but slightly improved safety toward HEK-293. This also confirmed that L-Pro is a safe ligand suitable for co-crystallization. In sharp contrast, both PD and PD-L-Pro showed much-reduced cytotoxicity against HEK-293 as compared to those of RSV and RSV-L-Pro. These results collectively suggested that PD and PD-L-Pro are potential anti-cancer reagents that are less toxic to normal cells, with PD-L-Pro further outperforming PD.

## 3. Materials and Methods

### 3.1. General

The resveratrol (RSV) and polydatin (PD) were purchased from Caobenyuan Biological Technology Co., Ltd. (Nanjing, China). The L-proline (L-Pro) was purchased from Aladdin (Shanghai, China). The Roswell Park Memorial Institute (RPMI) 1640 cell culture medium (for A549) and Dulbecco’s Modified Eagle Medium (DMEM) for HEK-293 were purchased from Bristol-Myers Squibb Trading Co. Ltd. (New York, NY, USA). The 3-(4,5-Dimethylthiazol-2-yl)-2,5-diphenyltetrazolium bromide (MTT) was obtained from Sigma (St. Louis, MO, USA). All the other chemicals were obtained directly from commercial sources and used as received. The ^1^H nuclear magnetic resonance (NMR) spectra were recorded on a Bruker DRX-400 spectrometer (Bruker, Ettlingen, Germany), and chemical shifts (δ) were referenced to the residual solvent peaks or internal standard TMS. FT-IR spectra were measured on a Thermo Scientific Nicolet iS50 FT-IR spectrometer (Thermo Fisher Scientific Co., Waltham, MA, USA) as KBr disks (400–4000 cm^−1^). Powder X-ray diffraction (PXRD) patterns were recorded on a Rigaku D/Max-2550PC micro-diffractometer (Rigaku Corporation, Tokyo, Japan). A rotating-anode Cu-target X-ray (λ = 1.5406 Å) was used, which was operated at 40 kV, 250 mA with a scanning range of 3.0 to 40.0° and a scanning speed of 5°/min^−1^ with an increasing step size of 0.02° and count time of 0.5–2 s. The TGA-DSC investigation was performed on a TA DSC Q100 differential filtering calorimeter (TA Instruments, New Castle, DE, USA) at a heating rate of 10 °C/min^−1^ under a nitrogen stream of 50 cm^3^/min^−1^. Ultraviolet–visible (UV–vis) spectra were collected on a Thermo Scientific Evolution 300 UV spectrometer (Thermo Scientific, Waltham, MA, USA). The cytotoxicity data were evaluated on a Bio-Rad Elisa Plate Reader 680 (Bio-Rad, Hercules, CA, USA).

### 3.2. Synthesis of Co-Crystals RSV-L-Pro and PD-L-Pro

The co-crystals were synthesized via the recrystallization method. RSV (228.25 mg, 1.0 mmol) and L-Pro (115 mg, 1.0 mmol) were dissolved in 3 mL of EtOH (100%), and the solution was stirred at 60 °C. The plate-shaped white crystals of RSV-L-Pro formed instantly.

PD (390 mg, 1.0 mmol) and L-Pro (115 mg, 1.0 mmol) were dissolved in 7.4 mL of EtOH (100%), and the solution was stirred at 60 °C for 30 min and then cooled to 25 °C. The plate-shaped colorless crystals of PD-L-Pro were formed after 24 h. 

Characterization data for L-Pro. ^1^H NMR (500 MHz, DMSO-*d*_6_) δ 3.65 (dd, J = 8.4, 5.8 Hz, 1H), 3.21 (dt, J = 12.4, 6.7 Hz, 1H), 3.01 (dt, J = 11.1, 7.6 Hz, 1H), 1.97 (ddq, J = 43.8, 12.6, 6.8, 5.9 Hz, 2H), 1.73 (ddq, J = 43.2, 12.8, 7.5, 6.9 Hz, 2H).

Characterization data for RSV. ^1^H NMR (500 MHz, DMSO-*d*_6_) δ 9.55 (s, 1H), 9.20 (s, 2H), 7.39 (d, J = 8.6 Hz, 2H), 6.98–6.79 (m, 2H), 6.75 (d, J = 8.6 Hz, 2H), 6.39 (s, 2H), 6.12 (t, J = 2.0 Hz, 1H).

Characterization data for RSV-L-Pro. ^1^H NMR (500 MHz, DMSO-d6) δ 9.27 (br, 7H), 7.38 (d, J = 8.7 Hz, 2H), 6.92 (d, J = 16.3 Hz, 1H), 6.80 (d, J = 16.3 Hz, 1H), 6.76 (d, J = 8.6 Hz, 2H), 6.38 (d, J = 2.0 Hz, 2H), 6.11 (t, J = 2.2 Hz, 1H), 3.81 (t, J = 7.2 Hz, 2H), 3.22 (dt, J = 11.2, 6.7 Hz, 2H), 3.05 (dt, J = 11.2, 7.3 Hz, 2H), 2.13–2.02 (m, 2H), 1.99–1.88 (m, 2H), 1.87–1.67 (m, 4H).

Characterization data for PD. ^1^H NMR (500 MHz, DMSO-*d*_6_) δ 9.49 (d, *J* = 69.7 Hz, 1H), 7.49–7.28 (m, 1H), 7.03 (d, *J* = 16.3 Hz, 1H), 6.92–6.66 (m, 2H), 6.33 (t, *J* = 2.1 Hz, 1H), 5.27 (d, *J* = 5.2 Hz, 1H), 5.04 (dd, *J* = 33.0, 5.1 Hz, 1H), 4.69–4.54 (m, 1H), 3.72 (ddd, *J* = 11.8, 5.2, 2.0 Hz, 1H), 3.48 (dt, *J* = 11.9, 6.1 Hz, 1H), 3.37–3.02 (m, 3H).

Characterization data for PD-L-Pro. ^1^H NMR (500 MHz, DMSO-*d*_6_) δ 9.68 (s, 1H), 9.53 (s, 1H), 8.72 (br, 6H), 7.39 (d, J = 8.6 Hz, 2H), 7.02 (d, J = 16.4 Hz, 1H), 6.86 (d, J = 16.4 Hz, 1H), 6.76 (d, J = 8.6 Hz, 2H), 6.72 (s, 1H), 6.56 (s, 1H), 6.33 (m, 1H), 5.55–4.89 (br, 2.83H), 4.79 (d, J = 7.7 Hz, 1H), 4.75–4.17 (br, 2.14H), 3.75–3.69(dd, J = 11.8, 2.1 Hz, 1H), 3.67-3.60 (dd, J = 8.7, 5.6 Hz, 3H), 3.52–3.47 (t, J = 5.9 Hz, 1H), 3.47–3.41 (q, J = 7.0 Hz, 2H), 3.35–3.12 (m, 7H), 3.05–2.96(m, 3H), 2.07–1.88 (m, 6H), 1.84–1.62 (m, 6H), 1.06 (t, J = 7.0 Hz, 3H).

### 3.3. Single-Crystal X-ray Crystallography

Single-crystal X-ray diffraction data were collected on a Bruker SMART APEX II diffractometer using graphite-monochromated Mo Kα radiation (λ = 0.71073 Å). The collected data were corrected for absorption with SADABS [39]. The structures were solved by direct methods and refined by full-matrix least-squares on *F*^2^ using SHELXTL-2016 [40]. Non-hydrogen atoms were refined anisotropically. The H atoms on the phenolic and alcoholic –OH and the cationic NH_2_ were placed in calculated positions either by HFIX instructions or with the Calc-OH program in the WinGX suite [41]. Crystallographic data for the crystal structures were deposited in the Cambridge Crystallographic Data Centre (CCDC) with supplementary numbers of 2090218 (RSV), 2090219 (RSV-L-Pro), 2090216 (PD), and 2090217 (PD-L-Pro). These data can be obtained free of charge from the CCDC via https://www.ccdc.cam.ac.uk/structures/.

### 3.4. Solubility and Dissolution Measurement

The solubility and dissolution studies of pure RSV, RSV-L-Pro, PD, and PD-L-Pro were conducted using a Thermo Logical Advancement UV–vis spectrometer in deionized water pH 7.0 solution at 37 °C. The concentration was calculated utilizing a standard curve, which was made by measuring the absorbance of different concentrations at their λ_max_.

The supersaturated solution was stirred at 150 rpm by magnetic stirring at 37 °C for 24 h, the suspension was filtered through a Whatman’s 0.45 mm syringe channel and diluted sufficiently, and the absorbance was measured at the λ_max_. For the dissolution rate, the sample was specifically poured into 250 mL of water and then stirred at 150 rpm for 2 h at 37 °C. At customary interims, 3 mL of the disintegration medium was pulled back and supplanted by a break-even with the volume of the new medium to preserve a steady volume. Each solution expelled was measured by the absorbance at λ_max_.

### 3.5. Cell Culture

The A549 and HEK-293 cell lines were purchased from American Type Culture Collection (Manassas, VA, USA). The cell lines were cultured in RPMI 1640 containing 10% fetal bovine serum (FBS) and 1% penicillin/streptomycin. Cells grew as a monolayer and were detached upon confluence using trypsin (0.5% *w/v* in PBS). The cells were harvested from the cell culture medium by incubating in trypsin solution for 3 min. The cells were centrifuged, and the supernatant was discarded. A 3 mL portion of serum-supplemented cell culture medium was added to neutralize any residual trypsin. The cells were re-suspended in serum-supplemented RPMI 1640 at a concentration of 5 × 10^4^ cells per 1 mL. Cells were cultured at 37 °C and 5% CO_2_ for MTT studies.

### 3.6. Cytotoxicity Evaluation by MTT Assay

The A549 and HEK-293 cells were seeded at a density of 1 × 10^4^ cells per well in 100 µL of complete RPMI 1640, and cultured for 16 h for attachment. The culture medium was then replaced by a serum-free medium containing various concentrations of pristine RSV, RSV-L-Pro, PD, and PD-L-Pro. After incubation for a period of 24 h, the MTT solution (100 μL, 0.5 mg/mL in serum-free RPMI 1640 medium) was added to replace the cell culture medium. After incubating the cells at 37 °C for 4 h, the MTT solution was removed and DMSO (100 μL) added to dissolve the insoluble formazan crystals, and the microplates were agitated for 5 min at a medium rate before the spectrophotometric measurement at a wavelength of 570 nm on a microplate reader. The untreated cells served as the 100% cell viability control, while the completely dead cells served as the blank. All experiments were carried out with four replicates. The relative cell viability (%) related to control cells was calculated using the formula below:

(1) $$V\%=\frac{{\left[A\right]}_{experimental}-{\left[A\right]}_{blank}}{{\left[A\right]}_{control}-{\left[A\right]}_{blank}}\times 100\%$$

where V% is the percentage of cell viability, [*A*]*_experimental_* is the absorbance of the wells culturing the treated cells, [*A*]*_blank_* is the absorbance of the blank, and [*A*]*_control_* is the absorbance of the wells culturing untreated cells.

## 4. Conclusions

Through co-crystallization with the naturally occurring L-Pro, two co-crystals of RSV-L-Pro and PD-L-Pro were successfully obtained. Solubility and solubility data showed that the eutectic form of PD and L-Pro exhibited better characteristics than the bulk drug. This could be because PD has one more natural glucose ring than RSV, which provided a wealth of hydrogen-bonding interactions, in addition to a channel structure, to promote the rapid diffusion of the solution medium. At the same time, PD-L-Pro showed better biological safety and certain anti-cancer potential. The idea of using biocompatible and non-toxic natural products in drug co-crystallization to synergistically improve the relevant properties of the bulk drug point to a new direction for drug formulations.

## Figures and Tables

**Table 1:** Summary of crystallographic data for RSV, PD, RSV-L-Pro, and PD-L-Pro.

Crystal Data	RSV	RSV-L-Pro	PD	PD-L-Pro
CCDC No.	2090218	2090219	2090216	2090217
Formula	C_14_H_12_O_3_	C_14_H_12_O_3_·2(C_5_H_9_NO_2_)	C_20_H_22_O_8_	C_20_H_22_O_8_·3(C_5_H_9_NO_2_)·C_2_H_6_O
M.W.	228.24	458.50	390.37	781.84
Crystal system	Monoclinic	Orthorhombic	Orthorhombic	Monoclinic
Space group	*P*2_1_/*c*	*P*2_1_2_1_2_1_	*P*2_1_2_1_2_1_	*C*_2_
Temperature (K)	170(2)	170(2)	170(2)	170(2)
*a* (Å)	4.3472(2)	5.55850(10)	7.3126(14)	39.40(3)
*b* (Å)	9.2128(4)	18.6566(4)	7.6691(14)	5.232(3)
*c* (Å)	26.6588(10)	21.5392(5)	33.051(5)	25.96(2)
*α* (°)	90	90	90	90
*β* (°)	92.953(2)	90	90	128.34(3)
*γ* (°)	90	90	90	90
*V* (Å^3^)	1066.26(8)	2233.67(8)	1853.5(6)	4198(6)
Z	4	4	4	4
*D*_c_/(g cm^−3^)	1.422	1.363	1.399	1.240
*F*(000)	480	976	824	1680
*μ* (mm^−1^)	0.100	0.833	0.916	0.804
Measured reflections	13,844	16,590	15,646	26,042
Independent reflections	1998	4001	3344	6730
Observed reflections	2359	4078	3386	7392
Flack parameter	/	0.05(7)	0.06(4)	−0.03(8)
*R*_int_	0.0397	0.0386	0.0400	0.0628
*R* (F^2^ > 2σ(F^2^))	0.0441	0.0413	0.0280	0.0584
wR(F^2^)	0.1124	0.1088	0.0725	0.1680
*GOF*	1.058	1.043	1.072	1.091
Parameters	154	301	259	508
Δρ_max_, Δρ_min_ (*e* Å^−3^)	0.254, −0.373	0.674, −0.261	0.166, −0.238	0.849, −0.412

## Data Availability

The data presented in this study are contained within this article and the Supplementary Materials.

## Supplementary Materials

The following are available online, Table S1 The characteristic diffraction peaks of RSV, RSV-L-Pro, PD and PD-L-Pro; Table S2 Hydrogen bonding tables for for RSV, RSV-L-Pro, PD, and PD-L-Pro; Figure S1 The 1H nuclear magnetic resonance (NMR) of L-Pro in DMSO-d6 solution; Figure S2 The 1H nuclear magnetic resonance (NMR) of RSV-L-Pro in DMSO-d6 solution. Figure S3 The 1H nuclear magnetic resonance (NMR) of PD-L-Pro in DMSO-d6 solution; Figure S4 TGA-DSC for the L-Pro.

## Sample Availability

Samples of the compounds RSV-L-Pro and PD-L-Pro are available from the authors.
