# Supplementary material for: Co-Crystals of Resveratrol and Polydatin with L-Proline: Crystal Structures, Dissolution Properties, and In Vitro Cytotoxicities
Source: Molecules. 2021 Sep 21;26(18):5722. doi: 10.3390/molecules26185722 (PMC8469398; doi:10.3390/molecules26185722)
Supplement: Supplementary file 1 [file molecules-26-05722-s001.zip › molecules-1364385-supplementary.pdf]

*Supplementary Materials for*

# Co-Crystals of Resveratrol and Polydatin with L-Proline: Crystal Structures, Dissolution Properties, and In Vitro Cytotoxicities

Yijie Lou, Kaxi Yu, Xiajun Wu, Zhaojun Wang, Yusheng Cui, Hanxiao Bao, Jianwei Wang, Xiurong Hu, Yunxi Ji * and Guping Tang *

**Table S1.** The characteristic diffraction peaks of RSV, RSV-L-Pro, PD and PD-L-Pro

| **RSV (2θ±0.1)°** | **RSV-L-Pro (2θ±0.1)°** | **PD (2θ±0.1)°** | **PD-L-Pro (2θ±0.1)°** |
|---|---|---|---|
| 6.56 | 6.22 | 5.28 | 4.30 |
| 10.06 | 6.60 | 10.60 | 5.64 |
| 13.20 | 8.16 | 11.72 | 6.74 |
| 16.34 | 9.42 | 12.30 | 8.98 |
| 19.15 | 10.26 | 12.60 | 10.40 |
| 19.44 | 12.46 | 13.94 | 12.94 |
| 19.90 | 13.12 | 16.06 | 14.90 |
| 20.28 | 14.72 | 16.62 | 17.08 |
| 22.12 | 15.46 | 16.82 | 17.30 |
| 22.32 | 16.36 | 17.56 | 17.82 |
| 23.04 | 17.02 | 17.96 | 18.06 |
| 23.38 | 18.76 | 18.44 | 18.64 |
| 23.58 | 20.62 | 19.76 | 19.32 |
| 24.10 | 21.04 | 20.02 | 20.38 |
| 25.22 | 21.70 | 21.34 | 22.00 |
| 27.79 | 22.58 | 21.94 | 22.30 |
| 28.26 | 24.66 | 22.26 | 22.80 |
| 28.50 | 25.08 | 23.14 | 23.84 |
| 28.97 | 26.10 | 23.62 | 24.14 |
| 30.66 | 26.44 | 24.28 | 24.46 |
| 31.46 | 28.92 | 25.10 | 25.78 |

**Table S2.** Hydrogen bonding tables for for RSV, RSV-L-Pro, PD, and PD-L-Pro

**RSV**

| D | H | A | d(D-H)/Å | d(H-A)/Å | d(D-A)/Å | D-H-A/° |
|---|---|---|---|---|---|---|
| O1 | H1A | O3[1] | 0.83 | 2.13 | 2.6788(17) | 123.9 |
| O1 | H1B | O2[2] | 0.83 | 1.88 | 2.6976(15) | 168.3 |
| O2 | H2A | O2[3] | 0.83 | 1.91 | 2.721(2) | 167.4 |
| O2 | H2B | O1[4] | 0.83 | 1.87 | 2.6976(15) | 175.5 |
| O3 | H3A | O3[5] | 0.83 | 1.85 | 2.683(2) | 176.8 |
| O3 | H3B | O1[6] | 0.83 | 1.85 | 2.6788(17) | 172.1 |

[1]1−X,−1/2+Y,1/2−Z; [2]+X,1/2−Y,−1/2+Z; [3]2−X,−Y,1−Z; [4]+X,1/2−Y,1/2+Z; [5]−X,1−Y,1−Z; [6]1−X,1/2+Y, 1/2−Z

**RSV-L-Pro**

| D | H | A | d(D-H)/Å | d(H-A)/Å | d(D-A)/Å | D-H-A/° |
|---|---|---|---|---|---|---|
| O2 | H2 | O5B[1] | 0.84 | 1.83 | 2.667(3) | 172 |
| O1 | H1 | O4A[2] | 0.84 | 1.85 | 2.673(3) | 166.8 |
| O3 | H3 | O4A[3] | 0.84 | 1.88 | 2.702(3) | 164.2 |
| N1A | H1AB | O4B[4] | 0.91 | 2.04 | 2.739(3) | 132.7 |
| N2A | H2AA | O5B[5] | 0.91 | 1.87 | 2.717(3) | 154.3 |

[1]1−X,−1/2+Y,1/2−Z; [2]3/2−X,1−Y,1/2+Z; [3]2−X,−1/2+Y,1/2−Z; [4]1/2−X,1−Y,−1/2+Z; [5]1+X,+Y,+Z

**PD**

| D | H | A | d(D-H)/Å | d(H-A)/Å | d(D-A)/Å | D-H-A/° |
|---|---|---|---|---|---|---|
| O1 | H1 | O5[1] | 0.84 | 1.8 | 2.6178(18) | 164.1 |
| O2 | H2 | O1[2] | 0.84 | 1.95 | 2.6899(19) | 146.6 |
| O5 | H5 | O7[3] | 0.84 | 1.97 | 2.7865(19) | 163.4 |
| O7 | H7 | O8[4] | 0.84 | 1.87 | 2.6906(17) | 166.5 |
| O8 | H8 | O2[5] | 0.84 | 1.82 | 2.6585(17) | 174.2 |

[1]1−1/2+X,1/2−Y,1−Z; [2]1/2+X,−1/2−Y,1−Z; [3]1+X,+Y,+Z; [4]−X,1/2+Y,1/2−Z; [5]−1+X,+Y,+Z

**PD-L-Pro**

| D | H | A | d(D-H)/Å | d(H-A)/Å | d(D-A)/Å | D-H-A/° |
|---|---|---|---|---|---|---|
| O6 | H6 | O7[1] | 0.84 | 2.08 | 2.857(4) | 153.1 |
| O7 | H7 | O9B | 0.84 | 1.9 | 2.742(4) | 177.8 |
| O8 | H8 | O10B[2] | 0.84 | 1.99 | 2.803(4) | 161.3 |
| O2 | H2 | O9A | 0.84 | 1.88 | 2.709(5) | 170.7 |
| N1A | H1AA | O10B[3] | 0.91 | 2.03 | 2.748(5) | 135.1 |
| N1A | H1AB | O10A[4] | 0.91 | 1.98 | 2.807(5) | 149.6 |
| O1 | H1 | O9C | 0.84 | 1.82 | 2.651(5) | 172 |
| N1C | H1CA | O10C[4] | 0.91 | 1.93 | 2.773(5) | 153.6 |
| N1C | H1CB | O9A[5] | 0.91 | 2.21 | 2.895(5) | 131.6 |
| O5 | H5A | O6[2] | 0.84 | 2.03 | 2.768(4) | 146.4 |
| O11A | H11A | O11B[6] | 0.84 | 2.54 | 3.18(4) | 134.2 |
| O11A | H11A | O11B | 0.84 | 2.29 | 2.95(3) | 135.6 |
| O11B | H11B | O11A | 0.84 | 2.18 | 2.95(3) | 151.8 |

[1]3/2−X,1/2+Y,1−Z; [2]+X,−1+Y,+Z; [3]1−X,−1+Y,1−Z; [4]+X,1+Y,+Z; [5]1/2−X,3/2+Y,−Z; [6]1−X,+Y,−Z

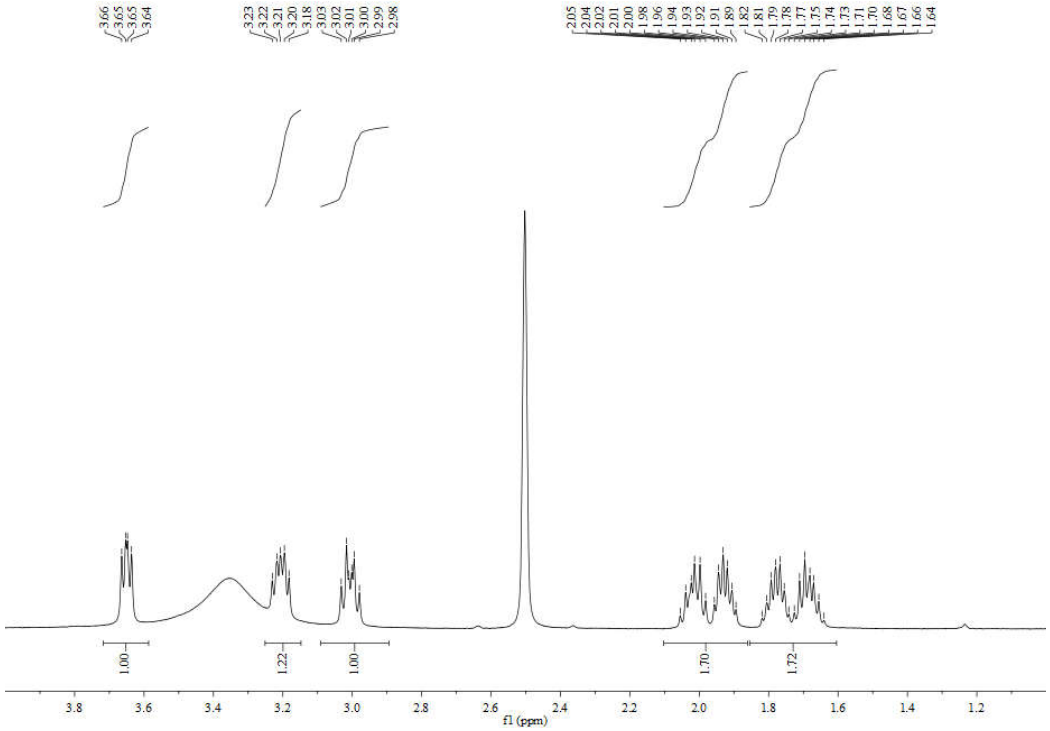

**Figure S1.** The $^1$H nuclear magnetic resonance (NMR) of L-Pro in DMSO-$d_6$ solution.

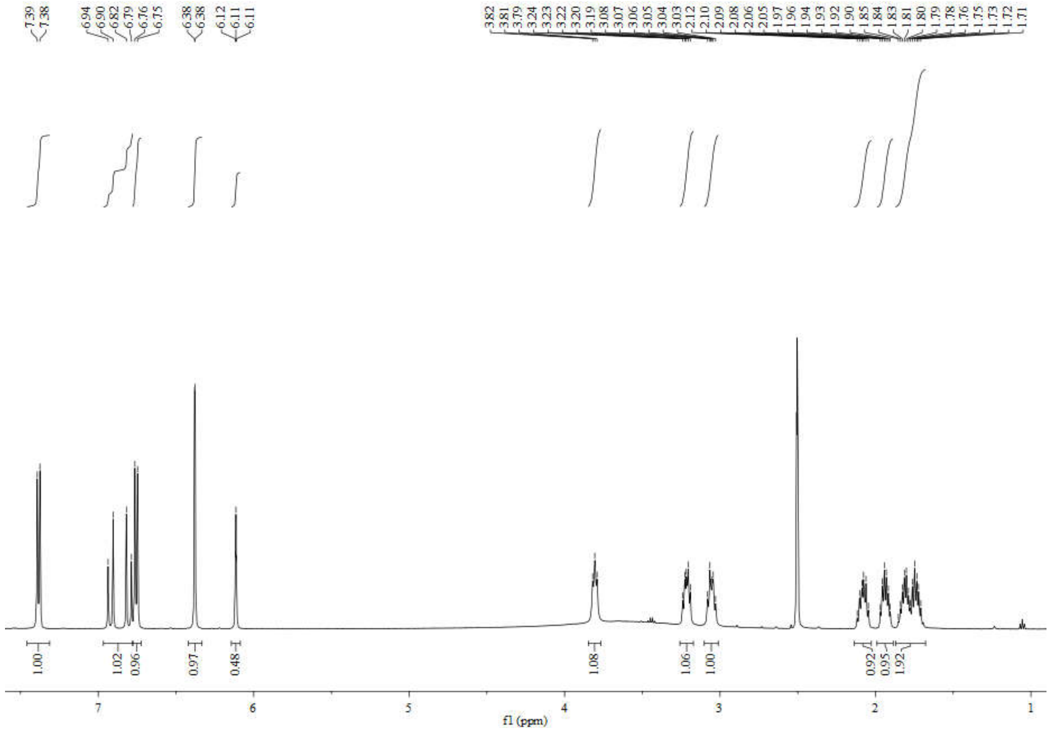

**Figure S2.** The $^1$H nuclear magnetic resonance (NMR) of RSV-L-Pro in DMSO-$d_6$ solution.

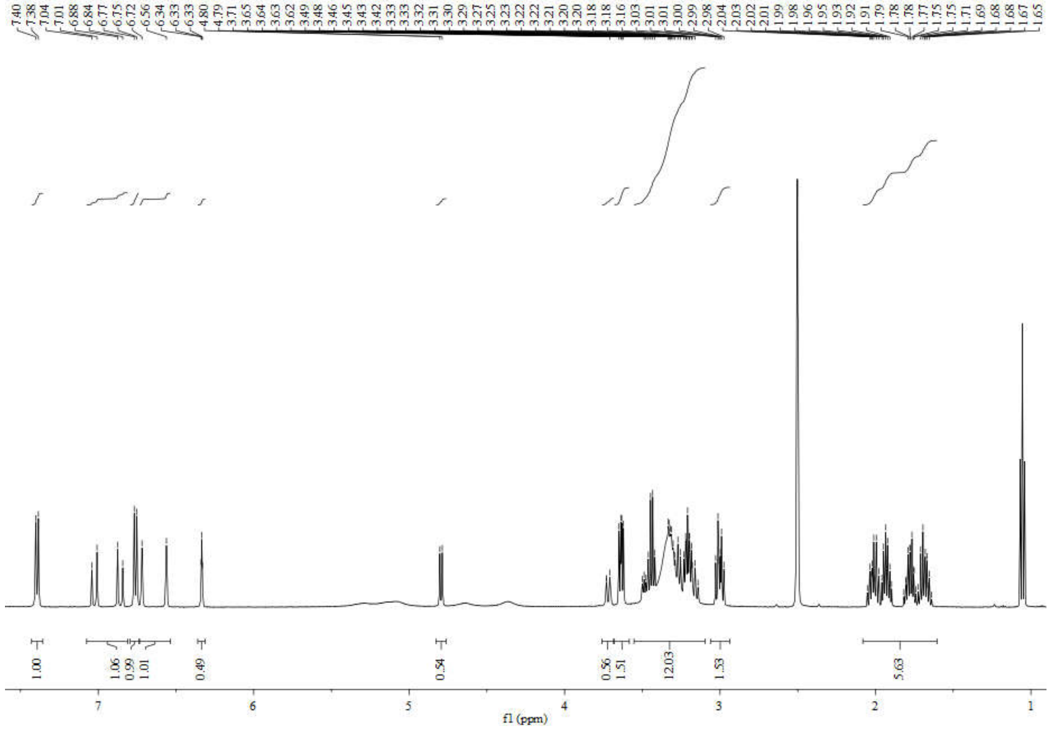

**Figure S3.** The $^1H$ nuclear magnetic resonance (NMR) of PD-L-Pro in DMSO-$d_6$ solution.

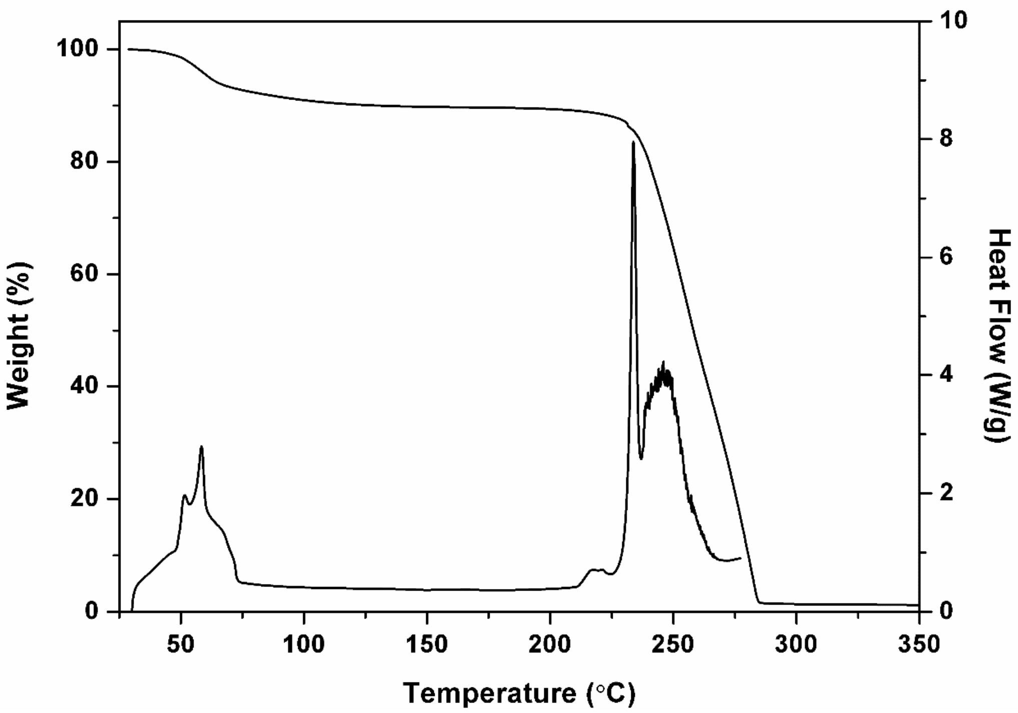


**Figure S4.** TGA-DSC for the L-Pro.
